# Development and validation of a digital burnout scale in artificial intelligence era

**DOI:** 10.3389/fpsyg.2025.1580422

**Published:** 2026-01-13

**Authors:** Lin Zhao, Jinxia Zhao, Ethan(Yi) Cao, Katherine(Ke) Li, Lei Pan, Yumei Zou, Xiaohan Sun

**Affiliations:** 1 College of Education, Linyi University, Linyi, China; 2 Teachers’ College of Vocational and Technology, Guangxi Normal University, Guilin, China; 3 School of Education, Taylor's University, Subang Jaya, Malaysia; 4 School of International Exchange, Hainan Medical University, Haikou, China; 5 Faculty of Arts and Social Sciences, Universiti Malaya, Kuala Lumpur, Malaysia; 6 School of Foreign Languages, Jiangxi Agricultural University, Nanchang, China

**Keywords:** interdisciplinary projects, pedagogical issues, evaluation methodologies, artificial intelligence, burnout

## Abstract

**Introduction:**

The rapid adoption of AI-driven digital technologies in higher education has intensified students’ exposure to digital demands, increasing the risk of digital burnout. Existing research lacks validated instruments that capture the multidimensional nature of digital burnout in AI-enhanced learning environments. This study aimed to develop and validate a comprehensive Digital Burnout Scale for college students, grounded in the Stressor–Strain–Outcome (SSO) model and Conservation of Resources (COR) theory.

**Methods:**

Using a multi-stage mixed-methods design, the study first conducted qualitative interviews to generate item pools, followed by large-scale survey data for quantitative validation. Exploratory and confirmatory factor analyses were performed to establish construct validity, supplemented by reliability testing and model comparison.

**Results and discussion:**

Findings supported a six-dimension structure of digital burnout: Digital Aging, Emotional Exhaustion, Cognitive Overload, Cognitive Dissonance, Digital Deprivation, and Behavioral Addictions. All dimensions demonstrated satisfactory convergent and discriminant validity, indicating strong psychometric robustness of the scale. The scale provides a reliable and theory-driven tool for assessing student digital burnout in the AI era. It offers practical value for educators and administrators seeking to identify high-risk groups and design targeted interventions.

## 1 Introduction

With the rapid advancement and widespread proliferation of Artificial Intelligence (AI) technology, humanity has ushered in the so-called “AI era” (Chiu et al., 2023). Virtually every facet of education, work, social interaction, and daily life has become increasingly reliant on intelligent algorithms and digital interfaces (Zhao et al., 2024). While this pervasive integration of technology enhances efficiency and convenience, it has also introduced a range of psychological and behavioral challenges, among which digital burnout stands out as a particularly salient issue (Da Silva et al., 2024). Digital burnout can be defined as a state of physical and emotional exhaustion resulting from prolonged work or excessive use of digital technologies and information tools in a digital environment (Aslan and Anandkumar, 2021). The problem was further exacerbated during the COVID-19 pandemic, as dependence on digital devices for work, learning, and socialization deepened significantly. Individuals spent almost all their waking hours online during lockdowns (Schmitt et al., 2021). Increased screen time, the normalization of multitasking, and the blurring of boundaries between work and personal life have contributed to heightened technostress and persistent “online fatigue” (Gregersen et al., 2023), substantially elevating the risk of digital burnout.

Traditional digital burnout is primarily triggered by information overload and screen dependency. Today’s digital environment has grown increasingly complex and highly personalized. The iterative advancement of artificial intelligence technologies has progressively shifted traditional “human-dominated tool-use scenarios” toward a “human–AI collaborative ecosystem” (Chan, 2023; Ouyang et al., 2022). Recent evidence suggests a dual-effect of generative AI on well-being, facilitating productivity and support while simultaneously elevating stress via algorithmic control, uncertainty, and overload (Büchi, 2024; Colder Carras et al., 2024; Dou and Sun, 2025). For instance, generative artificial intelligence blurs the boundaries of human–machine co-creation, and algorithmically tailored content based on user behavior and preferences can lead to unconscious prolongation of usage time, thereby reinforcing technology dependency and exacerbating feelings of burnout (Abdaljaleel et al., 2024; Issa et al., 2024). At the same time, pervasive concerns regarding data privacy and user autonomy may constitute a distinctive stressor specific to digital burnout in the age of artificial intelligence (Abulibdeh et al., 2024).

Studies have indicated that an excessive reliance on digital resources and AI-driven tools in educational contexts is closely linked to digital burnout (Duan and Zhao, 2024). Therefore, timely and thorough research on digital burnout is especially crucial and urgently needed in the current educational landscape. Although digital burnout is beginning to receive scholarly attention, there remains a lack of measurement tools specifically designed to assess digital burnout in the age of artificial intelligence. Existing burnout scales primarily focus on occupational and academic burnout (McEown et al., 2023; Salmela-Aro et al., 2009; Schaufeli et al., 2006), while other instruments, such as those measuring digital stress (Hall et al., 2021; Xie et al., 2023) or dependence on AI chatbots (Zhang et al., 2025), are largely grounded in traditional internet and social media environments. These tools are inadequate for capturing the novel characteristics of human–AI interaction emblematic of the current technological era. This gap underscores the necessity of developing a more contextually relevant measurement instrument. In response, this study aims to develop and validate a comprehensive scale specifically designed to evaluate digital burnout within AI-mediated contexts.

## 2 Conceptualization of “digital burnout” and literature review

Digital burnout has garnered increasing scholarly attention in recent years as a distinct form of psychological exhaustion associated with the pervasive integration of digital technology into various aspects of life. The concept originally stems from the classical psychological construct of “burnout.” Burnout is generally understood as a syndrome triggered by chronic, uncontrollable work stress, leading to the depletion of an individual’s internal resources and feelings of exhaustion or despondency (Freudenberger, 1974; Maslach and Jackson, 1981). It is typically characterized by emotional exhaustion, detachment, depersonalization, and a perceived inability to complete tasks (Leiter and Maslach, 2016). Digital burnout is often conceptually conflated with constructs such as digital stress, academic burnout, and problematic technology use. Academic burnout referred to a series of negative psychological manifestations such as anxiety, fatigue and depression in the learning process (Wang et al., 2021). It is often manifested as academic alienation, emotional exhaustion and reduced personal accomplishment (Salmela-Aro et al., 2009). Problematic technology use (PTU) refers to the excessive utilization of technologies, including the internet, video games and smartphones, as a central component of one’s daily life, which is accompanied by functional impairment and various emotional and behavioral issues (Elhai et al., 2020). Steele et al. (2020) define digital stress as the stress and anxiety arising from notifications and engagement with mobile and social media information and communication technologies.

Despite some conceptual overlap, fundamental distinctions remain between digital burnout and these related constructs. According to Aslan and Anandkumar (2021), digital burnout is a state of physical and emotional exhaustion resulting from prolonged work or excessive use of digital technology and information tools in a digital environment. In the age of artificial intelligence, the conceptual understanding of digital burnout has undergone further refinement. In this study, digital burnout in the AI era refers to a comprehensive psychological state manifested across cognitive, emotional, and behavioral dimensions, characterized by exhaustion, detachment, and reduced efficacy, which arises from the imbalance in human-machine relationships caused by individuals’ long-term exposure to persistent stressors in daily activities mediated by digital technologies, including generative AI. Erten and Özdemi̇R (2020) suggest that digital burnout encompasses three dimensions: digital aging, digital deprivation, and emotional exhaustion. Digital aging refers to the individual’s inability to balance the real world and the virtual world due to excessive use of digital platforms. Unlike general burnout or physical fatigue (Michielsen et al., 2003), the core characteristic of digital aging lies in the direct causal relationship between its symptomatology and digital technology usage behaviors. Digital deprivation refers to the physical and psychological discomfort experienced by a person when they are away from the digital environment. Emotional exhaustion is related to the depletion of emotional resources (Durmuş et al., 2022; Maslach and Leiter, 2016), and is typically characterized by fatigue, anxiety, and a sense of helplessness or being trapped due to the stress of a digital lifestyle.

A literature review indicates that digital burnout is closely associated with the negative psychosocial consequences resulting from individuals’ sustained and intensive interactions in digital technology environments, including generative AI (Duan and Zhao, 2024). It manifests as functional decline across emotional, physical, cognitive, and behavioral dimensions, which in turn gives rise to problems related to digital health as well as social adaptation (Ong et al., 2024). Furthermore, an empirical study by Zhu et al. (2025) points out that emotional exhaustion serves as a proximal risk factor for subjective well-being and exerts a significant negative predictive effect on it. Besides, continuous exposure to screens and digital stimuli can lead to physical issues such as eye strain, discomfort, and disruptions in sleep patterns. Over-reliance on digital technology may also lead to behavioral fatigue characterized by reduced attention span and difficulty concentrating on tasks at hand (Paterna et al., 2024). This could manifest as absenteeism or tardiness in professional settings, along with procrastination tendencies and social withdrawal. Ultimately, these lead to decreased productivity levels and diminished career motivation (Da Silva et al., 2024; Göldağ, 2022). The Turkish Language Agency (TLI) defines fatigue as a decline in an individual’s productivity in mental and physical activities due to factors such as work-related demands (Sirin et al., 2021). The term “behavioral fatigue” refers to an underlying psychological mechanism that impairs one’s ability to exhibit certain behaviors effectively (Harvey, 2020). Individuals experiencing fatigue often struggle with completing daily activities while feeling exhausted, weak, and lacking motivation.

Artificial intelligence tools contribute to the increasingly complex information environment. Information overload is a significant challenge faced by contemporary college students, leads to cognitive overload. Various terms have been employed in psychological and social science research to describe this phenomenon, such as information overload, technology overload, or digital overload, to capture the psychological consequences arising from exposure to an overwhelming number of news sources or distress caused by content on information platforms. Additionally, concerns about communication overload or social overload mainly revolve around excessive use of social media (Schmitt et al., 2021). Iskander (2019) used the term “cognitive overload” to describe the various forms of overload experienced when the amount of information received exceeds its processing capacity due to excessive use of digital tools. Furthermore, the ethical issues, factual problems, and risks of misinformation spread associated with AI tools can also lead to cognitive dissonance. According to cognitive dissonance theory, cognitive dissonance is the discomfort that an individual experiences when they hold two or more contradictory beliefs, attitudes, or behaviors within themselves. The theory of cognitive dissonance holds that people naturally pursue the consistency and harmony of their inner cognition. When cognitive dissonance exists, individuals will feel psychological discomfort and pressure (Harmon-Jones and Mills, 2019; Van Lange et al., 2012).

Recent research has increasingly emphasized the necessity of cross-cultural validation of burnout assessment instruments (De Beer et al., 2024; Schaufeli et al., 2023). For instance, the Burnout Assessment Tool (BAT) has been adapted and validated across diverse cultural contexts, including Brazil, Australia, and the United States, confirming its robust cross-regional applicability and underscoring the importance of employing culturally sensitive and contextually adapted tools in burnout evaluation (Redelinghuys and Morgan, 2023).

Amidst the profound transformation of human-computer interaction by artificial intelligence technologies, although scholarly attention has begun to focus on the manifestations and antecedents of digital burnout, two critical research gaps persist in the field. First, a comprehensive theoretical framework capable of systematically elucidating the distinctive features of AI and their mechanistic impacts on digital burnout remains underdeveloped. Second, there is a notable absence of specialized assessment tools with demonstrated reliability and validity designed specifically to evaluate digital burnout within AI-mediated contexts. These theoretical and methodological limitations substantially constrain both a deeper understanding of psychological adaptation in human-AI collaborative settings and the development of effective interventions, underscoring an imperative for focused academic inquiry and dedicated research efforts.

This study integrates the Stressor-Strain-Outcome (SSO) model (Koeske and Koeske, 1993) and Conservation of Resources (COR) theory (Hobfoll, 1989), to systematically explain its formation mechanism. The SSO model divides the stress-handling process into three components: stressors, strain, and outcomes (Ali Ather et al., 2024; Zolkepli et al., 2024). It provides a clear causal pathway, where AI technological characteristics (e.g., algorithmic opacity, personalized overload) constitute stressors, trigger immediate psychological strain (e.g., cognitive overload, emotional exhaustion), and ultimately lead to the stable outcome of digital burnout. From a motivational perspective, COR theory further elaborates this process by emphasizing that individuals have a fundamental motivation to maintain, protect, and acquire their existing resources as well as gain new ones (Halbesleben et al., 2014). Sustained interactions in AI environments may result in the continuous loss of individuals’ key resources (e.g., attention, sense of autonomy, cognitive and emotional energy), and burnout will occur when resource recovery is hindered or attempts to acquire new resources fail.

**Figure 1:**
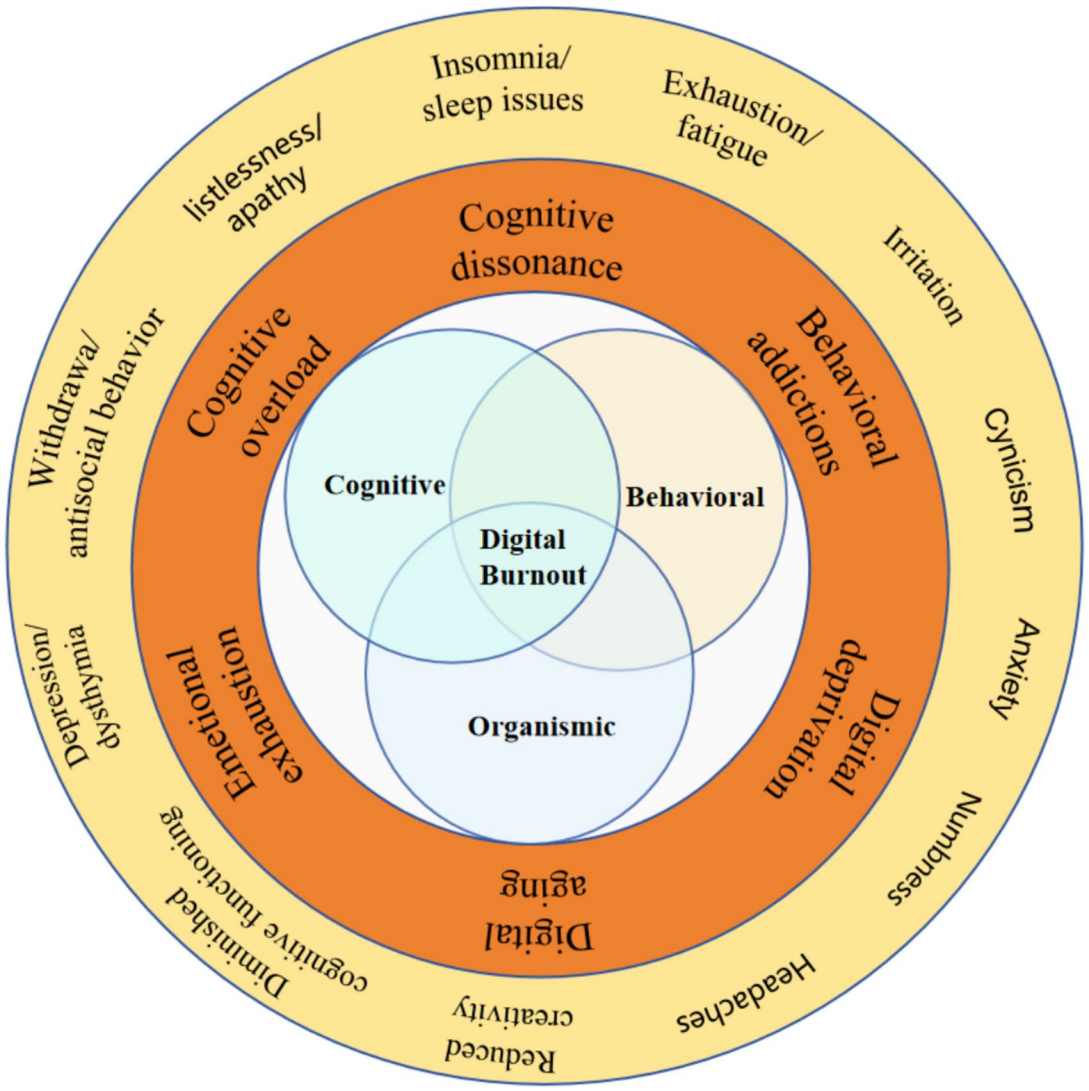
conceptual framework.

Symptoms of burnout encompass various manifestations (Yi et al., 2024), including insomnia/sleep disturbances, exhaustion/fatigue, irritability, cynicism, anxiety, numbness, headaches, reduced creativity and cognitive functioning, depression, withdrawal/antisocial behavior, listlessness/apathy among others (Leiter and Maslach, 2016; Maslach et al., 2001; Maslach and Jackson, 1981). According to burnout symptoms and previous research, we conceptualize digital burnout from three dimensions: organismic, cognitive, and behavioral. These dimensions, when combined, encompass various constructs such as digital deprivation, emotional exhaustion, cognitive overload, cognitive dissonance, digital aging, behavioral addictions, etc. Ultimately, we have constructed a conceptual framework for digital burnout as outlined in Figure 1.

## 3 Methods

The participants of this study consist of students from three universities in China and one university in Malaysia, who possess extensive experience in utilizing digital technologies. This group was selected due to their high engagement with digital technologies and strong reliance on them for both academic pursuits and daily life activities.

Snowball sampling was employed during the interview stage, while a combination of a purposive sampling and convenience sampling was used during the questionnaire survey stage to ensure content relevance and accuracy.

This study is divided into three distinct phases.

Phase 1: Qualitative Interviews. In the qualitative phase of this study, semi-structured interviews were conducted with 15 college students aged 18–25 to explore their experiences of digital burnout in AI-enhanced learning environments. Each interview lasted approximately 30–45 min, was conducted online, and audio-recorded with participant consent. All recordings were transcribed verbatim and anonymized prior to analysis. To ensure methodological rigor and reproducibility, we adopted a six-phase thematic analysis process as outlined by Braun and Clarke (2006). Two researchers (the first author and the corresponding author) independently conducted open coding using NVivo 14 to identify meaningful excerpts related to digital burnout. These codes were iteratively grouped into preliminary themes, which were subsequently refined through constant comparison and team discussions. A final coding framework was established, which comprises six core themes: Digital Aging, Emotional Exhaustion, Cognitive Overload, Cognitive Dissonance, Digital Deprivation, and Behavioral Addiction. To assess inter-coder reliability, both researchers independently coded 20% of the transcripts (*n* = 6), selected randomly. Cohen’s Kappa was calculated at 0.84, indicating excellent agreement (Cohen, 1960). All discrepancies were resolved through consensus discussions, and the final codebook was documented and made available via OSF (Open Science Framework) for transparency and replicability.

Phase 2: Exploratory Factor Analysis (EFA). Item generation combined literature pooling, student interviews, and expert review; content validity quantified via I-CVI/S-CVI. EFA used minres+oblimin with eigen>1/scree/parallel analysis; item retention loading ≥ 0.50, h^2^ ≥ 0.40, Δ ≥ 0.20. All indicators were measured on 7-point Likert scales (ordered categories approximating interval level). We examined distributional assumptions (univariate skewness/kurtosis and Mardia’s multivariate kurtosis) and observed mild deviations from normality. First, we randomly distributed 50 questionnaires among a voluntary student group to refine the developed scale. Through analysis of these collected questionnaires, an initial round of revisions was conducted to enhance their quality. Second, further validation of the revised questionnaire was performed through surveys completed by over 130 participants. EFA was executed using Jamovi software as an open-source tool.

Phase 3: Confirmatory Factor Analysis (CFA). Sample-size adequacy was considered according to McNeish and Wolf (2023), which links required N to the expected loading magnitude, number of indicators per factor, and number of factors. Given six factors with ~6 indicators each and typical loadings around 0.70, our N (279) falls within the recommended range for stable CFA estimation. CFA used covariance-based SEM (CB-SEM) in SmartPLS 4.1; we report χ^2^, df, χ^2^/df, CFI, RMSEA, and SRMR; reliability by Cronbach’s *α* and composite reliability (ρc); validity by AVE and HTMT < 0.85. In this phase, online questionnaires were employed to collect data for confirmatory factor analysis (CFA) via partial least squares structural equation modeling (PLS-SEM). The primary objective was to evaluate the structural validity and internal consistency of various items and dimensions within the questionnaire while also verifying if the structure identified in Phase two could be confirmed, aiming for favorable model fit indices. To effectively conduct CFA, we utilized CB-SEM technology through SmartPLS 4.1 software.^1^

We ensured that all participants in this study were informed about their voluntary participation and anonymity, as well as their right to withdraw at any point during the process. Both interviewees and questionnaire respondents were above 18 years of age. To protect participant privacy, we employed iFlytek’s voice transcription software during interviews to instantly convert spoken conversations into written transcripts without revealing personal identities. The questionnaire utilized a seven-point Likert scale ranging from “strongly disagree” to “strongly agree” for assessing participants’ perspectives. Additionally, demographic and academic background information such as gender and major were collected anonymously.

This study was approved by the academic committee of first and corresponding author’s universities. We have collected primary data with the permission and supervision of concerned teacher and institute and all research was performed in accordance with relevant guidelines.

## 4 Scale development and data analysis

### 4.1 Scale development process

We followed established guidance for scale development and reporting (DeVellis and Thorpe, 2021; Hinkin, 1998; Worthington and Whittaker, 2006) and aligned our procedures with the COSMIN standards (Prinsen et al., 2018) and APA JARS (JARS-Quant; Appelbaum et al., 2018) to enhance transparency and reproducibility. To generate the initial item pool for a digital burnout scale among college students, we conducted a targeted literature review spanning burnout, digital burnout, digital stress, cognitive overload, and fatigue (Burleson et al., 2023; Da Silva et al., 2024; Iskander, 2019; Maslach and Leiter, 2016; Sirin et al., 2021). We then extracted candidate items from the questionnaire compiled by Erten and Özdemi̇R (2020) and, to address conceptual gaps not covered by existing scales, developed several supplemental items tailored to AI-mediated study and work contexts.

To ensure the comprehensiveness of the scale in encompassing all relevant aspects, qualitative interviews were conducted with a target group of 30 college students to explore their experiences of burnout associated with digital devices and platforms. These interviews were conducted in their native language to facilitate unrestricted expression of thoughts and emotions. Open-ended questions were utilized during these interviews to gain insights into how students managed prolonged periods of digital device usage and symptoms related to burnout.

Based on analysis of coding techniques through grounded theory, a number of subject-scale items were preliminarily identified. Duplicates or similar items were subsequently excluded after comparison with existing studies. The novel items that emerged from the interviews but had not been covered by existing scales were considered unique and retained if mentioned by more than three participants. Through this iterative process, in which researchers involved in data analysis procedures reached a consensus, a final set comprising 38 scale items was obtained, which included new data obtained during interviews. The results are presented in Table 1.

**Table 1:** Developing scale items.

Items
X1: Staring at digital screen for a long time causes dry and sore eyes.
X2: After prolonged use of AI devices, I feel muscle tension and soreness in my shoulders, neck, or back.
X3: After continuously using AI for multitasking, I feel dizzy and mentally and physically exhausted.
X4: It easily disrupts my schedule, leading to difficulty falling asleep or poor sleep quality.
X5: Irregular work and rest will make my immunity decline and often get sick.
X6: Using digital devices for entertainment, I feel like time is being wasted, my mood is unstable, and I tend to feel more irritable mentally.
X7: Prolonged use of smartphones can lead to anxiety and guilt, and the more anxious I become, the harder it is to put down the phone.
X8: When various digital platforms send irrelevant messages, I feel irritable and prone to impulsive reactions.
X9: If the digital platform keeps pushing similar information that has been browsed or information that is not of great interest, I become bored.
X10: In the information environment augmented by artificial intelligence, I often feel exhausted and anxious due to the presence of false information.
X11: When multiple AI platforms frequently provide an excess of erroneous and inaccurate information, I feel bewildered and at a loss, struggling to discern the authenticity of certain pieces of information.
X12: The omnipresent advertisements tempt me with various shopping needs, making me feel annoyed and exhausted.
X13: The abundance of learning resources on AI platforms requires considerable time and effort to select the most valuable content, which increases the time cost and learning pressure.
X14: The vast amount of knowledge available online is sometimes too much to absorb, having a feeling my brain is about to explode, leading to a loathing of learning and a desire to vomit, which affects my study progress.
X15: Over-reliance on AI may constrain my thought processes and narrow my perspective, leading to a gradual weakening of my critical thinking abilities.
X16: Prolonged use of digital devices leads to my distraction, which makes me easily distracted and difficult to concentrate when I study.
X17: Unconsciously, I compare the colorful life on the internet with my own, leading to dissatisfaction with my real life, a sense of disparity and frustration, resulting in emotional imbalance, breakdowns, and anxiety.
X18: Digital learning inevitably makes me aware of the learning progress and achievements of different learners around the world. Comparing myself with them invisibly increases my anxiety, depression, and academic pressure.
X19: The application of AI greatly expands my understanding of the world and increases the amount of information, which invisibly makes me feel anxious and inferior.
X20: Seeing photos of others people’s good-looking can trigger anxiety about my own appearance.
X21: If I do not actively contact the information provided by AI-driven devices, I will worry about being out of touch with the current society, feeling compelled to accept digital media.
X22: Digital life has a significant impact on my personal views and even values, affecting my subjective judgments, such as my career choices.
X23: Everyone can express their unique perspectives through digital platforms, and some of these viewpoints continuously reshape my values and affect my emotions.
X24: When encountering topics with strong controversies online, the views expressed their influence on me, leading to internal conflicts and affecting some of my own value judgments.
X25: Without access to AI-driven devices, such as during exams, I become more anxious and flustered.
X26: I feel uneasy and have a sense of loss and loneliness when I do not have Internet connection or offline.
X27: I feel naked and insecure when I do not have my digital devices (phone, tablet, computer etc.…) with me.
X28: I check my digital media all the time, afraid of missing any message. If I do not, I feel anxious or unaccustomed.
X29: I feel very tired when I get out of the state of using AI-driven devices.
X30: I unconsciously imitate the behavior of some digital platform bloggers.
X31: I am often addicted to entertainment software unrelated studies, which weakens my focus on academics, reduces learning efficiency, and hinders my learning progress.
X32: I gradually lose myself in the online world, losing interest and motivation in studying.
X33: If I need to complete a learning task, I will directly use AI to search for information and refer to it, lacking my own contemplation.
X34: The things in the physical world can rarely arouse my interest, and it is difficult for me to sit quietly and read the paper books I am interested in.
X35: I am accustomed to turning on digital devices and unwilling to stop using them, even if there is no specific use for them. If I do not, I will feel a sense of emptiness and do not know what to do.
X36: I use digital devices excessively, neglecting relatives and friends, and my relationships and communications with people have been weakened.
X37: My excessive dependence on digital devices has led to a decrease in my offline social activities, and my face-to-face relationships have weakened.
X38: I feel that interpersonal communication on the Internet is more relaxed, and may turn to online virtual social activities.

In this phase of the research, we developed the initial version of Digital burnout scale, an acronym for Scale for Digital Burnout. This tool and framework not only build upon but also expand the digital burnout framework proposed by Erten and Özdemi̇R (2020). Digital burnout scale incorporates fundamental elements derived from the previous framework, encompassing digital aging, digital deprivation, and emotional exhaustion explored in prior studies while further extending its scope.

### 4.2 Exploratory factor analysis

To establish and improve the reliability and validity of the scale items, we initially conducted a pilot study on a limited sample size (*n* = 50). During this phase, students completed the questionnaire in the presence of research team members who recorded and discussed any ambiguous questions or items. Based on student feedback, certain items were rephrased or adjusted to ensure clarity and precision in question presentation. Subsequently, we administered online surveys to students in classrooms and invited the same group of pre-test students to complete the revised survey online for evaluating its functionality and item clarity.

Based on the feedback received, we determined that further extensive data collection should be conducted to test the scale preparation. Ultimately, a total of 130 valid questionnaires were successfully retrieved. To assess the suitability of the questionnaire data for factor analysis, we computed the Kaiser-Meyer-Olkin (KMO) measurements and Bartlett’s test sphericity (refer to Table 2). The KMO value for all items is 0.66, which exceeds the recommended threshold of 0.5, indicating that our sample is suitable for exploratory factor analysis (EFA). Additionally, Bartlett’s sphericity test yielded a result of 1773 with a *p*-value less than 0.001, suggesting a significant correlation in our data. This demonstrates its appropriateness for factor analysis according to Hair et al. (2019).

**Table 2:** Test of KMO and Bartlett’s.

Bartlett’s test of sphericity	χ^2^	df	*p*
KMO measure of sampling adequacy	1773	703	< 0.001
		MSA	
	Overall	0.66	

**Figure 2:**
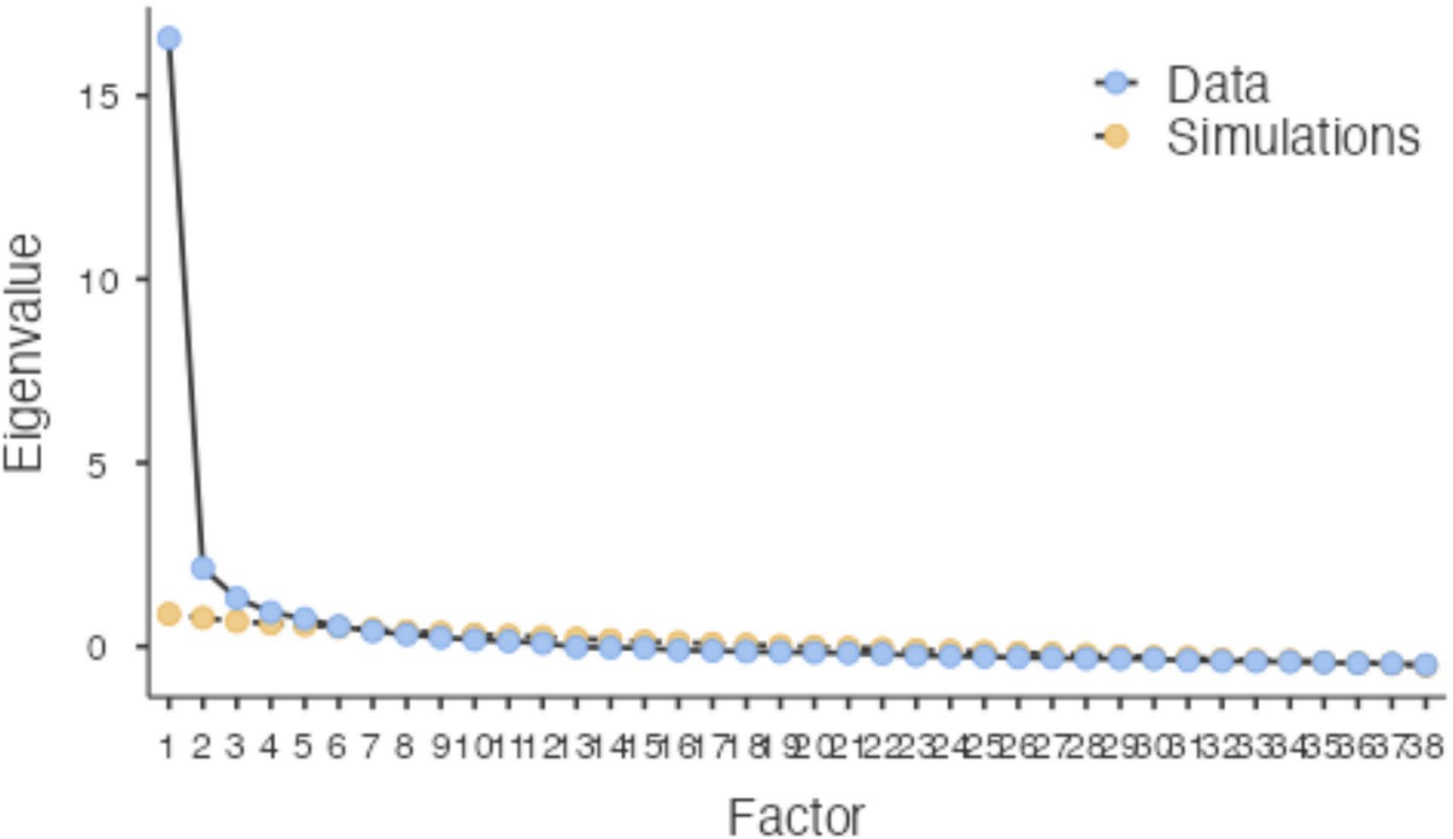
Scree plot of eigenvalues for 1 through 38 potential factors.

In the exploratory factor analysis (EFA) phase, the minimum residual extraction method was used for factor extraction, and the oblimin rotation method (*δ* = 0) was applied to address inter-factor correlations. The number of factors was determined based on three integrated criteria: ① eigenvalues > 1.00; ② inflection points the Scree plot (see Figure 2); ③ parallel analysis (95th percentile). The findings of this analysis are presented in Table 3. Items were retained based on the following standards: communality h^2^ ≥ 0.40, primary factor loading ≥ 0.50, and cross-loading difference ≥ 0.20 (Hair et al., 2021). Accordingly, item X30 (“I imitate the behavior of bloggers”) was deleted as it failed to meet the above thresholds—with excessively high uniqueness (h^2^ = 0.507) and all primary loadings < 0.50. After deletion, the Cronbach’s *α* coefficient increased from 0.847 to 0.851, confirming its exclusion. A total of 37 items were finally retained, with the six factors explaining 62.4% of the total variance collectively. The majority of the remaining items exhibit factor loadings greater than or close to 0.50, indicating a clear factor structure that meets psychometric requirements (Hair, 2019).

**Table 3:** Factor loadings.

Item	Factor 1	Factor 2	Factor 3	Factor 4	Factor 5	Factor 6	Uniqueness
X1	0.70						0.48
X2	0.68						0.45
X3	0.89						0.26
X4	0.67						0.44
X5	0.64						0.46
X6	0.52						0.45
X7	0.49						0.44
X31		0.71					0.25
X32		0.60					0.36
X33		0.70					0.35
X34		0.64					0.31
X35		0.78					0.33
X8	0.35		0.50				0.36
X9			0.56				0.39
X10			0.77				0.33
X11			0.55				0.47
X12			0.69				0.28
X13			0.49				0.51
X14			0.50	0.32			0.43
X15			0.49				0.41
X16			0.50				0.41
X25				0.59			0.42
X26				0.73			0.27
X27		0.34		0.49			0.43
X28				0.50			0.41
X29				0.51			0.43
X36				0.60			0.34
X37				0.52			0.44
X38				0.49	0.3		0.43
X17					0.50		0.34
X18			0.31		0.55		0.30
X19					0.71		0.19
X20					0.51		0.34
X21						0.55	0.39
X22						0.57	0.36
X23						0.53	0.39
X24						0.50	0.43
X30							0.51

Primary loadings ≥ 0.50 (guideline). Several borderline items (loadings around 0.49) were retained given strong content validity and acceptable discrimination (Δ ≥ 0.20) and communality (h^2^ ≥ 0.40).

Regarding multiple comparisons in EFA, we did not apply blanket Bonferroni or FDR corrections because (a) such procedures are often overly conservative for exploratory factor retention and can undermine construct coverage (Perneger, 1998), and (b) our multi-criteria decision rules (primary loading ≥ 0.50, cross-loading difference *Δ* ≥ 0.20, communality h^2^ ≥ 0.40) already constrain false positives at the item level (Hair et al., 2019). We therefore prioritized theory-consistent retention under these pre-specified rules and report the full item-level statistics to enable independent appraisal.

Fit evaluation followed Brown (2015) and Kline (2023), with cutoffs referenced to Hu and Bentler (1999), and sample size adequacy considered per McNeish and Wolf (2020). Based on the aforementioned EFA results, we identified six factors, each corresponding to distinct dimensions of digital burnout among college students: Digital Aging (Factor 1), Behavioral Addiction (Factor 2), Emotional Exhaustion (Factor 3), Digital Deprivation (Factor 4), Cognitive Overload (Factor 5), and Cognitive Dissonance (Factor 6). After excluding Item X30, 37 items were retained, most showed primary loadings ≥ 0.50 or borderline (0.47–0.49) consistent with recommended thresholds for exploratory work (Hair, 2019). We then conducted a confirmatory factor analysis (CFA) to evaluate the plausibility of this six-factor structure.

### 4.3 Confirmatory factor analysis

**Figure 3:**
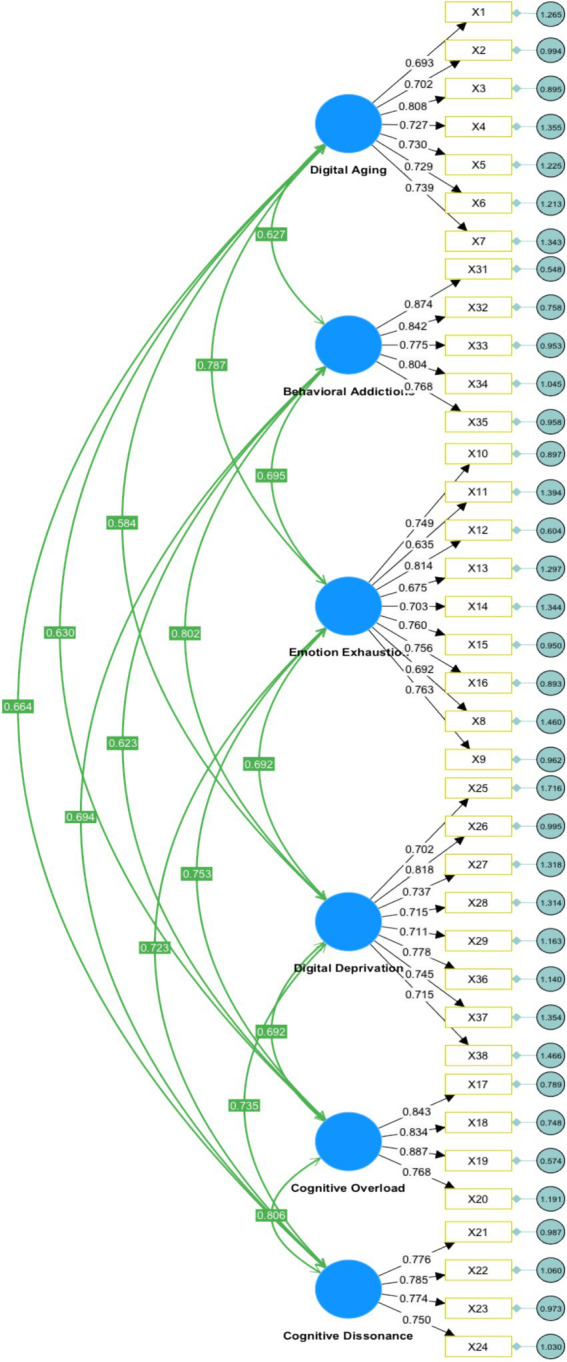
Result of CFA.

In the final stage, we estimated the six-factor measurement model using covariance-based SEM (CB-SEM) in SmartPLS 4.1 (CB-SEM module). We reported χ^2^/df, CFI, and RMSEA. Fit evaluation followed Brown (2015) and Kline (2023). We interpreted CFI and RMSEA using Hu and Bentler’s guidelines (e.g., CFI ≥ 0.95, RMSEA ≤ 0.06; Hu and Bentler, 1999), and considered sample-size adequacy per McNeish and Wolf (2020). Results are summarized in Tables 4–7 and Figure 3. CFA was used to evaluate how well the observed indicators represent the hypothesized latent constructs and, together with reliability and validity evidence, to assess the robustness of the measurement model.

**Table 4:** Loading of CFA.

	Digital aging	Emotion exhaustion	Digital deprivation	Cognitive overload	Cognitive dissonance	Behavior addictions
X1	0.69					
X2	0.70					
X3	0.81					
X4	0.73					
X5	0.73					
X6	0.73					
X7	0.74					
X8		0.69				
X9		0.76				
X10		0.75				
X11		0.64				
X12		0.81				
X13		0.68				
X14		0.70				
X15		0.76				
X16		0.75				
X25			0.70			
X26			0.82			
X27			0.74			
X28			0.72			
X29			0.71			
X36			0.78			
X37			0.75			
X38			0.72			
X17				0.844		
X18				0.834		
X19				0.886		
X20				0.769		
X21					0.776	
X22					0.787	
X23					0.773	
X24					0.749	
X31						0.874
X32						0.842
X33						0.775
X34						0.804
X35						0.778

**Table 5:** Construct reliability and validity.

	Cronbach’s alpha (standardized)	Composite reliability (rho_c)	Average variance extracted (AVE)
Behavior addictions	0.91	0.91	0.66
Cognitive dissonance	0.85	0.86	0.60
Cognitive overload	0.90	0.90	0.70
Digital aging	0.89	0.89	0.54
Digital deprivation	0.91	0.91	0.55
Emotion exhaustion	0.91	0.91	0.53

**Table 6:** Discriminant validity-HTMT.

	Behavioral addictions	Cognitive dissonance	Cognitive overload	Digital aging	Digital deprivation	Emotional exhaustion
Behavioral addictions						
Cognitive dissonance	0.70					
Cognitive overload	0.65	0.82				
Digital aging	0.62	0.67	0.64			
Digital deprivation	0.81	0.75	0.71	0.58		
Emotional exhaustion	0.69	0.72	0.77	0.79	0.72	

**Table 7:** Model fit.

	Estimated model	Null model
Chi-square	2015.82	8524.91
Number of model parameters	89.00	37.00
Number of observations	279.00	n/a
Degrees of freedom	614.00	666.00
*p* value	0.00	0.00
ChiSqr/df	3.51	12.80
RMSEA	0.080	0.21
GFI	0.82	n/a
CFI	0.90	n/a
SRMR	0.06	n/a
AIC	2329.92	n/a
BIC	2653.09	n/a

According to Hair et al. (2019), a crucial consideration in confirmatory factor analysis (CFA) is the magnitude of the factor loading, which indicates the alignment between a factor and its underlying construct when there is high convergent validity. It is commonly recommended that standardized loading estimates should be at least 0.5 or ideally 0.7 or higher. In our measurement model, item loadings were generally high (most ≥ 0.70; all ≥ 0.50 or borderline), as reported in Table 4, supporting convergent validity.

Convergent validity can also be assessed by considering reliability, as different reliability coefficients may yield slightly varied estimates. However, in structural equation modeling (SEM) models, a marginally different construct reliability (CR) value is commonly employed. A high construct reliability score of 0.70 indicates the presence of internal consistency, suggesting that all measures consistently represent the same underlying construct. In our study, all constructs demonstrated strong construct reliability with Cronbach’s alpha and rho_c values exceeding 0.7 (refer to Table 5).

Average variance extracted (AVE) serves as a convergence measure, representing the average variance extracted from items loading on a specific construct. It is calculated as the mean squared factor loading or average communality. An AVE value equal to or greater than 0.5 indicates satisfactory convergence. In our current study, all constructs exhibited an AVE greater than 0.5, indicating satisfactory convergence (refer to Table 5).

Discriminant validity refers to the extent of differentiation between a construct or variable and other constructs or variables, providing evidence for the unique nature of a construct. In addition to relying on Cross Loading and Fornell-Larcker criterion, Henseler et al. (2015) introduced the heterotrait-monotrait (HTMT) ratio of correlations as an alternative measure. Issues related to discriminant validity arise when HTMT values exceed acceptable thresholds. A value exceeding 0.90 suggests inadequate discriminant validity; however, for more distinct conceptual constructs, it is recommended to adopt a lower threshold value such as 0.85 in order to be more conservative in assessing discriminant validity. In our current research, all HTMT values are below 0.85, indicating satisfactory discriminant validity for each construct (refer to Table 6).

The model fit output obtained from testing the confirmatory factor analysis (CFA) model is presented in Table 7 in this analysis. The CFI value of 0.9 and RMSEA value of 0.08 indicate a favorable model fit, thus suggesting strong agreement between the observed data and the hypothesized model. It is worth noting that the statistically significant ChiSqr/df value of 3.5 further supports these findings.

Accordingly, we estimated the CFA using covariance-based SEM (CB-SEM) in SmartPLS 4.1, yielding Huber–White robust standard errors and scaled χ^2^ statistics. Missing data were handled via the Expectation–Maximization (EM) algorithm implemented in SmartPLS 4.1, and listwise deletion was avoided to reduce bias and loss of power.

The finalized 37-item scale (six constructs: Digital Aging, Behavioral Addiction, Emotional Exhaustion, Digital Deprivation, Cognitive Overload, and Cognitive Dissonance) comprises 37 items (see Appendix) and showed adequate overall fit, though marginal on some indices (ChiSqr/df = 3.51; CFI ≈ 0.90; RMSEA ≈ 0.08; Table 7). Given the theory-constrained specification and high standardized loadings (most ≥ 0.70; Table 4), we retained the six-factor solution.

## 5 Discussion

This study introduces a preliminary Digital Burnout Scale, which aims to expand upon previous instruments by incorporating six distinct dimensions: digital aging, emotional exhaustion, cognitive overload, cognitive dissonance, digital deprivation, and behavioral addiction. Unlike prior tools that were often brief, our scale is informed by data derived from interviews with college students in real digital environments and adapted to reflect current technological trends. Moreover, the scale has undergone initial quantitative verification in selected settings, suggesting enhanced reliability and validity.

Cognitive and Emotional Dimensions of Digital Burnout. Current research highlights that digital burnout manifests not only through negative physical and emotional effects but also through cognitive overload and cognitive dissonance. This phenomenon is largely attributed to the unprecedented volume of information in today’s digital environment (Cao and Sun, 2018). Individuals are often bombarded with information from various channels, such as personalized push notifications and ubiquitous advertising, which may lead to feelings of boredom and fatigue during multitasking. Some studies have shown that prolonged exposure to high cognitive load can induce anxiety states (Cezar and Maçada, 2023), potentially resulting in decision-making errors and dysfunction.

Physical and Mental Manifestations of Digital Burnout. Current findings suggest that digital burnout is associated with notable physical fatigue, including symptoms such as eye pain, migraines, shoulder and neck pain, insomnia, and possibly increased susceptibility to illness. These results align with previous research (Romero-Rodríguez et al., 2023), indicating that excessive exposure to media information may disrupt sleep patterns, impair concentration, and compromise immune system functionality, potentially leading to stress-induced illnesses. Digital burnout may also manifest as mental exhaustion, particularly when individuals spend extended time in front of screens, experience information overload, or struggle to disconnect from their devices. This can contribute to anxiety, guilt, emotional instability, irritability, and impulsivity. Emotional exhaustion is considered a fundamental aspect of burnout (Moon and Hur, 2011; Seidler et al., 2014), and our study further emphasizes its relevance within the context of digital burnout.

Social and Comparative Aspects of Digital Burnout. The internet provides a broad perspective but also facilitates social comparisons of appearance, life status, learning progress, and academic performance with idealized online content (Alkis et al., 2017). Such comparisons can often lead to dissatisfaction with one’s real-life self, potentially creating a sense of disparity and resulting in anxiety, depression, and feelings of inferiority complex (Jiang and Ngien, 2020). This potential influence may extend to altering individuals’ career choices and values, partly due to the curated, filtered, and embellished nature of social media content. Selective exposure and interpretation of information are thought to tend to focus on glamorous aspects rather than providing a comprehensive or objective understanding.

Behavioral Addictions and their Implications. The current study further proposes behavioral addictions as a potential manifestation of digital burnout. Individuals experiencing exhaustion may struggle to complete daily activities, feeling drained both physically and mentally, and may experience a lack of motivation (Yaakoubi et al., 2024). Among college students, excessive reliance on digital devices may lead to decreased attention span during learning sessions, diminished interest and motivation for studying, and impaired learning efficiency and progress (Tülübaş et al., 2023). Additionally, immersion in the online world can negatively impact personal relationships by reducing offline social engagements and neglecting communication with relatives and friends (Basel et al., 2020). These behavioral manifestations highlight the potential multifaceted impact of a digital lifestyle on individual learning outcomes, interpersonal relationships, and emotional states.

Beyond merely identifying the dimensions of digital burnout, our findings allow for a synthesized characterization of its unique qualities in the AI era. Unlike traditional burnout, AI-facilitated digital burnout appears to be distinguished by three core features: (1) Algorithmic mediation. Burnout stressors stem not only from workload but are more prominently orchestrated and amplified by opaque AI systems such as personalized information feeds (Büchi et al., 2019) and algorithmic management (Gagrčin et al., 2024). (2) Novelty of cognitive load. Cognitive exhaustion arises not merely from information overload, but also from the persistent need to evaluate the authenticity of AI-generated content, mitigate algorithmic biases, and address ethical ambiguities (Tafesse et al., 2024). (3) Specificity of resource depletion. It involves the exhaustion of unique resources, such as cognitive autonomy (eroded by algorithmic decision-making) and privacy self-efficacy (depleted by constant vigilance toward datafication; Khetawat and Steele, 2023), while simultaneously exhibiting the alienated feature of “functional dependence” (Huang et al., 2024), a shift from “recreational dependence” to direct reliance on AI for learning. These multifaceted traits highlight the necessity of developing the scale in this study.

The Digital Burnout Scale introduced in this study is designed to serve as a potential scientific and practical assessment tool, aiming to provide a potentially reliable quantitative measure of digital burnout among college students in the context of artificial intelligence. This finding is consistent with Erten and Özdemi̇R (2020) perspective that digital burnout is a multifaceted concept encompassing not only exhaustion from operating digital technology but also physical, emotional, and social challenges arising during the processing of digital information. Our proposed scale and preliminary findings may contribute to the understanding and assessment of digital burnout in contemporary digital environments.

## 6 Conclusion

The current study has developed and validated the Digital Burnout Scale, a novel tool designed to measure the extent of burnout among college students in digital learning environments within the context of the artificial intelligence (AI) era. This scale builds upon and significantly expands previous research on digital burnout by integrating current technological advancements and offering a comprehensive conceptual framework for understanding the multifaceted components of digital burnout in educational settings enhanced by AI.

Initially, an exploratory factor analysis was conducted on the Digital Burnout Scale, yielding a preliminary version with six factors and 38 items. This scale was further refined and validated through confirmatory factor analysis (CFA). The final version of the Digital Burnout Scale, detailed in the Appendix, comprises six subscales with a total of 37 items: Digital Aging (7 items), Emotional Exhaustion (9 items), Cognitive Overload (4 items), Cognitive Dissonance (4 items), Digital Deprivation (8 items), and Behavioral Addictions (5 items).

Digital Aging reflects the physical and mental fatigue stemming from the overuse of digital devices in our increasingly digitalized world. Symptoms include ocular discomfort, migraines, nausea, and even vomiting due to prolonged screen exposure, as well as physical issues such as shoulder and neck pain, spinal discomfort, and hair loss. It can also lead to mental health problems like dizziness, cognitive decline, and exhaustion, which disrupt sleep patterns and immune function. Excessive use of digital devices for entertainment can lead to feelings of wasted time and emotional instability. Long-term smartphone use can potentially cause anxiety and guilt, and it also increases the difficulty of breaking usage habits.

Emotional Exhaustion, characterized by the depletion of emotional resources, is a common phenomenon in the digital age. It manifests as fatigue and disinterest in the constant information flow from digital platforms, with repetitive social media content leading to monotony. The prevalence of false information online increases anxiety and skepticism. Moreover, the saturation of online advertising contributes to mental and emotional exhaustion. The abundance of online learning resources increases the stress of selecting quality materials, negatively impacting learning outcomes. The overwhelming amount of knowledge available online can create an aversion to learning. Furthermore, AI-driven personalized recommendations can constrain individual thinking and impair critical thinking abilities over time.

Cognitive Overload occurs when individuals are exposed to more information than they can cognitively process, often due to excessive use of digital tools. This can lead to negative psychological and emotional outcomes, such as dissatisfaction and frustration from comparing one’s life to others’ online portrayals, and anxiety and depression from perceiving oneself as falling short compared to global peers. The vast array of information available online can create cognitive overload, leading to anxiety and low self-esteem, particularly when individuals encounter idealized social media images that trigger self-worth anxieties.

Cognitive Dissonance arises from holding contradictory beliefs, attitudes, or behaviors, exacerbated by exposure to diverse information from electronic devices. Concerns about missing out on digital information can lead to feelings of disconnection and pressure to accept digital media content even when it is uncomfortable. Digital life significantly impacts personal opinions and values, influencing judgments and beliefs. Cognitive dissonance occurs when online information conflicts with personal values, and the values conveyed through electronic products can profoundly affect mental well-being. Low educational levels can increase the likelihood of being exposed to biases and misinformation, which skews user values and negatively impacts mental health. Internal conflicts from engaging with negative content can lead to confusion, stress, and mental health issues.

Digital Deprivation results from overreliance on digital platforms and refers to the discomfort of being disconnected from the digital environment. It manifests as panic and anxiety during device-free periods, insecurity, a sense of being lost, and loneliness without internet access. The absence of electronic devices leaves individuals feeling exposed and vulnerable. Fear of missing out leads to anxiety and unease, and overuse of digital devices causes fatigue and disconnection from reality. Over-dependence on electronic devices weakens interpersonal relationships and communication skills, as face-to-face interactions deteriorate giving way to virtual networking.

Behavioral Addiction involves compulsive engagement in activities like gambling or social media use despite negative consequences, distinct from substance dependence. College students are especially susceptible to behavioral addiction due to excessive use of entertainment software, which undermines their academic focus and learning efficiency. This addiction promotes dependence on instant gratification rather than critical thinking and deep learning, replacing interest and motivation with apathy. Students rely on search engines or AI for answers rather than contemplating problems, thus weakening critical thinking and problem-solving skills. The captivating nature of digital devices makes it difficult to detach, even without a specific purpose, as users become accustomed to constant stimulation.

Digital technology is indispensable for college students, however, excessive reliance or improper use can lead to digital burnout, affecting brain development and overall well-being, and potentially hindering learning outcomes. Addressing digital burnout in higher education is crucial, as students heavily depend on digital devices and internet resources. This study’s comprehensive scale for assessing digital burnout among college students has been validated for both scientific and practical value, providing a reliable tool for measuring digital burnout levels. By identifying and quantifying digital burnout, the study aids educators in developing personalized teaching strategies and learning resources to alleviate digital burnout and foster a healthy digital learning environment. It also offers new insights into human resource development by examining the impact of digital technology on human psychology and behavior. Future research should explore interventions to mitigate digital burnout and further investigate the long-term effects of AI and digital technology on student well-being and academic performance.

## 7 Implications

This study addresses a growing concern in higher education during the artificial intelligence era by developing and validating a scale designed to measure digital burnout among college students. As digital learning becomes increasingly integral to education, students are navigating significant changes in learning methods while also facing potential negative effects. The proposed Digital Burnout Scale aims to offer a practical instrument that could help students better recognize their own levels of digital burnout, understand its complexities, and potentially access more tailored support to promote both academic engagement and mental well-being.

Furthermore, this research highlights the importance of supporting mental health in digitally saturated learning environments. By integrating the Stressor-Strain-Outcome (SSO) model and Conservation of Resources (COR) theory, the study seeks to provide a theoretical framework for understanding how technology-related stressors may impact psychological resources and contribute to burnout. This theoretical integration could guide the design of targeted interventions, including support services and coping strategies, that may help reduce digital burnout, enhance digital literacy, and foster healthier learning habits.

The findings may also offer valuable insights for educators and administrators. By utilizing this scale, educational practitioners could gain a deeper understanding of students’ digital learning experiences, which might support the development of adaptable teaching strategies and learning resources. These efforts, in turn, could contribute to more supportive digital learning environments and enhanced student well-being.

## 8 Limitations and future research

This study has made initial progress in developing a digital burnout scale for college students, but several limitations should be acknowledged. First, the sample was culturally concentrated, consisting primarily of Chinese students with a small number of Malaysian students. As a result, the factor structure and manifestation of “digital burnout” captured by the scale may reflect culture-specific norms regarding technology use, academic pressure, and emotional expression. Caution is therefore needed when generalizing these findings to students in other cultural or educational contexts. Second, the mixed-methods design relied on non-probability sampling in both qualitative and quantitative phases, which limits generalizability and may introduce self-selection and response biases (e.g., social desirability and interpretive bias). These issues should be considered when interpreting the findings. Third, the majority of research on artificial intelligence in education is cross-sectional, capturing only a snapshot of the current state and hindering the identification of the developmental trajectory and contributing factors of digital burnout. Fourth, the ethical and privacy considerations of artificial intelligence in education, particularly in relation to digital burnout, have not been thoroughly examined. Fifthly, the impact of institutional support in mitigating digital burnout has not been sufficiently investigated.

Future research directions could encompass: (i) validating the scale in more diverse cultural contexts (e.g., Western and African settings) and testing cross-cultural measurement invariance; (ii) recruiting larger and more socio-demographically diverse samples, clearly reporting participants’ characteristics (e.g., gender, age, and socio-economic background) and examining potential group differences in digital burnout; (iii) strengthening mixed-methods integration by explicitly linking qualitative themes with quantitative factors, such as using joint-display tables that map qualitative codes onto specific items and latent dimensions; (iv) employing a broader range of research methodologies, including experimental and longitudinal designs, to investigate the determinants of digital burnout and its associations with academic performance and mental health, as well as the long-term impact of AI use; (v) engaging in deeper discussions of the ethical and privacy implications of AI in education, particularly in relation to digital burnout; and (vi) evaluating the effectiveness of different forms of support, such as mental health services, digital detox programs, and workload management strategies, in alleviating digital burnout.

## Data Availability

The original contributions presented in the study are included in the article/supplementary material, further inquiries can be directed to the corresponding authors.

## Footnotes

1
https://www.smartpls.com/release_notes
