# Supplementary material for: Development and validation of a digital burnout scale in artificial intelligence era
Source: Front Psychol. 2026 Jan 13;16:1580422. doi: 10.3389/fpsyg.2025.1580422 (PMC12836882; doi:10.3389/fpsyg.2025.1580422)
Supplement: Supplementary file 1 [file Data_Sheet_1.docx]

**Appendix**: Scale instrument

| DIMENSIONS | | SCALE ITEMS |
| --- | --- | --- |
| Digital Burnout Scale | Behavior Addictions | X1: I am often addicted to entertainment software unrelated studies, which weakens my focus on academics, reduces learning efficiency, and hinders my learning progress. |
|  |  | X2: I gradually lose myself in the online world, losing interest and motivation in studying. |
|  |  | X3: If I need to complete a learning task, I will directly use AI to search for information and refer to it, lacking my own contemplation. |
|  |  | X4: The things in the physical world can rarely arouse my interest, and it is difficult for me to sit quietly and read the paper books I am interested in. |
|  |  | X5: I am accustomed to turning on digital devices and unwilling to stop using them, even if there is no specific use for them. If I don’t, I will feel a sense of emptiness and don’t know what to do. |
|  | Cognitive Dissonance | X6: If I do not actively contact the information provided by AI-driven devices, I will worry about being out of touch with the current society, feeling compelled to accept digital media. |
|  |  | X7: Digital life has a significant impact on my personal views and even values, affecting my subjective judgments, such as my career choices. |
|  |  | X8: Everyone can express their unique perspectives through digital platforms, and some of these viewpoints continuously reshape my values and affect my emotions. |
|  |  | X9: When encountering topics with strong controversies online, the views expressed their influence on me, leading to internal conflicts and affecting some of my own value judgments. |
|  | Cognitive Overload | X10: Unconsciously, I compare the colorful life on the internet with my own, leading to dissatisfaction with my real life, a sense of disparity and frustration, resulting in emotional imbalance, breakdowns, and anxiety. |
|  |  | X11: Digital learning inevitably makes me aware of the learning progress and achievements of different learners around the world. Comparing myself with them invisibly increases my anxiety, depression, and academic pressure. |
|  |  | X12: The application of AI greatly expands my understanding of the world and increases the amount of information, which invisibly makes me feel anxious and inferior. |
|  |  | X13: Seeing photos of others people’ s good-looking can trigger anxiety about my own appearance. |
|  | Digital Aging | X14: Staring at digital screen for a long time causes dry and sore eyes. |
|  |  | X15: After prolonged use of AI devices, I feel muscle tension and soreness in my shoulders, neck, or back. |
|  |  | X16: After continuously using AI for multitasking, I feel dizzy and mentally and physically exhausted. |
|  |  | X17: It easily disrupts my schedule, leading to difficulty falling asleep or poor sleep quality. |
|  |  | X18: Irregular work and rest will make my immunity decline and often get sick. |
|  |  | X19: Using digital devices for entertainment, I feel like time is being wasted, my mood is unstable, and I tend to feel more irritable mentally. |
|  |  | X20: Prolonged use of smartphones can lead to anxiety and guilt, and the more anxious I become, the harder it is to put down the phone. |
|  | Digital Deprivation | X21: Without access to AI-driven devices, such as during exams, I become more anxious and flustered. |
|  |  | X22: I feel uneasy and have a sense of loss and loneliness when I don’t have Internet connection or offline. |
|  |  | X23: I feel naked and insecure when I do not have my digital devices (phone, tablet, computer etc.…) with me. |
|  |  | X24: I check my digital media all the time, afraid of missing any message. If I don’t, I feel anxious or unaccustomed. |
|  |  | X25: I feel very tired when I get out of the state of using AI-driven devices.  X26: I use digital devices excessively, neglecting relatives and friends, and my relationships and communications with people have been weakened. |
|  |  | X27: My excessive dependence on digital devices has led to a decrease in my offline social activities, and my face-to-face relationships have weakened. |
|  |  | X28: I feel that interpersonal communication on the Internet is more relaxed, and may turn to online virtual social activities. |
|  | Emotion Exhaustion | X29: When various digital platforms send irrelevant messages, I feel irritable and prone to impulsive reactions. |
|  |  | X30: If the digital platform keeps pushing similar information that has been browsed or information that is not of great interest, I become bored. |
|  |  | X31: In the information environment augmented by artificial intelligence, I often feel exhausted and anxious due to the presence of false information. |
|  |  | X32: When multiple AI platforms frequently provide an excess of erroneous and inaccurate information, I feel bewildered and at a loss, struggling to discern the authenticity of certain pieces of information. |
|  |  | X33: The omnipresent advertisements tempt me with various shopping needs, making me feel annoyed and exhausted. |
|  |  | X34: The abundance of learning resources on AI platforms requires considerable time and effort to select the most valuable content, which increases the time cost and learning pressure. |
|  |  | X35: The vast amount of knowledge available online is sometimes too much to absorb, having a feeling my brain is about to explode, leading to a loathing of learning and a desire to vomit, which affects my study progress. |
|  |  | X36: Over-reliance on AI may constrain my thought processes and narrow my perspective, leading to a gradual weakening of my critical thinking abilities. |
|  |  | X37: Prolonged use of digital devices leads to my distraction, which makes me easily distracted and difficult to concentrate when I study. |
